# Suicide Prevention in Patients with Severe Mental Disorders

**DOI:** 10.1192/j.eurpsy.2022.138

**Published:** 2022-09-01

**Authors:** P. Courtet

**Affiliations:** University Hospital Montpellier, Emergency Psychiatry And Acute Care, Montpellier, France

**Keywords:** psychological pain,, immediate interventions, antidepressants,, suicide prevention

## Abstract

Until 2016, only few interventions were supposed to work in suicide prevention: restriction of access to lethal means, school-based universal prevention, treatment of depression and ensuring chain of care. Then, despite the multiplication of the therapeutic strategies for psychiatric disorders during the last decades, the incidence of suicide has not substantially decreased. Among several hypotheses, we proposed that suicidal depression is a specific form of depression, less responsive to antidepressants, carrying a high suicide risk, which deserves specific interventions. During the last decade, few controlled studies have been performed in at risk patients with short term reduction of the risk of suicide as a main objective, and the interest for old drugs such as lithium and clozapine remains. Recent data allow to propose that a new era is coming with evidence-based strategies of suicide prevention that should lead to change the way we deal with suicidal patients. Importantly, most efforts to develop interventions have moved to a perspective that suicide- specific treatments are necessary in addition to interventions for primary psychiatric disorders. By formulating the hypothesis that suicidal patients present a dysregulated response to social adversity based on specific brain areas associated with psychological pain, relying to opioidergic, immune and glutamatergic systems. Last, due to the difficult management of suicidal patients, innovative psychosocial interventions should be implemented for patients in suicidal crises and including safety planning, coordination of care, brief contact using phone calls. We have probably more solutions than ever to prevent suicide.

**Disclosure:**

No significant relationships.

